# Chemokine receptor CXCR4 regulates CaMKII/CREB pathway in spinal neurons that underlies cancer-induced bone pain

**DOI:** 10.1038/s41598-017-04198-3

**Published:** 2017-06-21

**Authors:** Xue-Ming Hu, Hui Zhang, Heng Xu, Hai-Long Zhang, Li-Ping Chen, Wen-Qiang Cui, Wei Yang, Wen Shen

**Affiliations:** 1 Department of Pain Medicine, Affiliated Hospital of Xuzhou Medical University, Xuzhou, Jiangsu 221002 China; 2 Jiangsu Province Key Laboratory of Anesthesiology and Jiangsu Province Key Laboratory of Anesthesia and Analgesia Application Technology, Xuzhou Medical University, Xuzhou, Jiangsu 221002 China; 3 Department of Integrative Medicine and Neurobiology, Academy of Integrative Medicine, School of Basic Medical Sciences, Fudan University, Shanghai, 200032 China

## Abstract

We previously demonstrated that the chemokine receptor CXCR4 plays an important role in cancer-induced bone pain by activating spinal neurons and glial cells. However, the specific neuronal mechanism of CXCR4 signaling is not clear. We further report that CXCR4 contributes to the activation of the neuronal CaMKII/CREB pathway in cancer-induced bone pain. We used a tumor cell implantation (TCI) model and observed that CXCR4, p-CaMKII and p-CREB were persistently up-regulated in spinal neurons. CXCR4 also co-expressed with p-CaMKII and p-CREB, and mediated p-CaMKII and p-CREB expression after TCI. Intrathecal delivery of CXCR4 siRNA or CaMKII inhibitor AIP2 abrogated TCI-induced pain hypersensitivity and TCI-induced increase in p-CaMKII and p-CREB expression. Intrathecal injection of the principal ligand for CXCR4, SDF-1, promoted p-CaMKII and p-CREB expression in naive rats, which was prevented by post-administration of CXCR4 inhibitor Plerixafor or PLC inhibitor U73122. Plerixafor, U73122, or AIP2 also alleviated SDF-1-elicited pain behaviors. Intrathecal injection of CXCR4 siRNA significantly suppressed TCI-induced up-regulation of NMDAR1 mRNA and protein, which is a known gene target of CREB. Collectively, these results suggest that the CaMKII/CREB pathway in spinal neurons mediates CXCR4-facilitated pain hypersensitivity in cancer rats.

## Introduction

Cancer-induced bone pain (CIBP), resulting from primary tumor of bone or tumor metastasis to bone, remains one of the most common and distressing types of cancer pain^1^. Recent epidemiological studies have demonstrated that approximately half or more of patients with metastatic or advanced osteocarcinoma experience daily moderate to severe cancer pain, which severely disrupts their quality of life^2^. Cancer pain symptoms, generally consisting of pathological breakthrough (or incident) pain and ongoing pain, are inadequately managed by using conventional analgesics and therapies, which are partially because of our current poor understanding of the specific mechanisms of this pain^3^.

The chemokine C-X-C motif receptor 4 (CXCR4)^4^, which belongs to G protein-coupled receptors (GPCRs), is the primary receptor of stromal-derived factor-1 (SDF-1, also known as CXCL12). Recent studies have demonstrated that CXCR4 chemokine signaling contributes to the development and maintenance of chronic pain hypersensitivity, which is characterized by mechanical allodynia and heat hyperalgesia. The underlying cellular mechanism is implicated in neuronal sensitization and glial activation, because CXCR4 is located in both spinal neurons and glial cells^5–10^. Our previous results showed that spinal CXCR4 mediates CIBP generation and c-Fos up-regulation in spinal cord^11^. However, the detailed intracellular molecular mechanism of CXCR4 in spinal neurons underlying CIBP is not clear.

Ca^2+^/calmodulin-dependent protein kinase II (CaMKII) is abundant in postsynaptic densities of spinal and supraspinal neurons, and is involved in synaptic plasticity and long-term potentiation (LTP) in nociceptive sensitization^12, 13^. Cyclic adenosine monophosphate response element-binding protein (CREB) is a primary downstream target of CaMKII that plays a cardinal role in the generation of hyperalgesia by increasing CREB-dependent and pain-related gene expression, such as c-Fos^14^, COX-2 (cyclooxygenase-2)^15^, NK-1 (neurokinin-1)^16^, BDNF (brain-derived neurotrophic factor)^17^, and so on. Considerable evidence suggests that GPCRs, including chemokine receptors, activate phospholipase (PLC), which catalyses the hydrolysis of phosphatidylinositol 4,5-bisphosphate (PIP2) to produce inositol 1,4,5-trisphosphate (IP3). IP3 releases intracellular Ca^2+^ (Ca^2+^i) from the endoplasmic reticulum (ER) by binding to IP3 receptors (IP3Rs)^18, 19^. The increased concentration of Ca^2+^i is necessary to activate the CaMK family. Therefore, we reasonably hypothesized that CXCR4 facilitates CIBP development by activating CaMKII/CREB signaling in spinal neurons.

The current study used the well-characterized tumor cell implantation (TCI)-induced cancer pain rat model to investigate the neuronal mechanism of CXCR4 signaling pathway in the pathophysiology of CIBP. We demonstrated that CXCR4, phospho-CaMKII (p-CaMKII) and phospho-CREB (p-CREB) were functionally induced in the spinal cord dorsal horn and contributed to CIBP in TCI rats. We observed that intrathecal (i.t.) injection of exogenous SDF-1 triggered pain behaviors by inducing CXCR4-mediated CaMKII/CREB phosphorylation in spinal neurons of naive rats. We clarified the hypothesis that spinal CXCR4 promoted CIBP by up-regulating N-methyl-D-aspartate receptor subunit 1 (NMDAR1) mRNA and protein expression, which is a CREB-dependent and pain-related gene target. Spinal blockade of the CXCR4-CaMKII/CREB pathway may be a potential analgesic therapy for cancer pain management.

## Results

### Up-regulation and co-localization of CXCR4, p-CaMKII and p-CREB in spinal neurons after TCI

**Figure 1:**
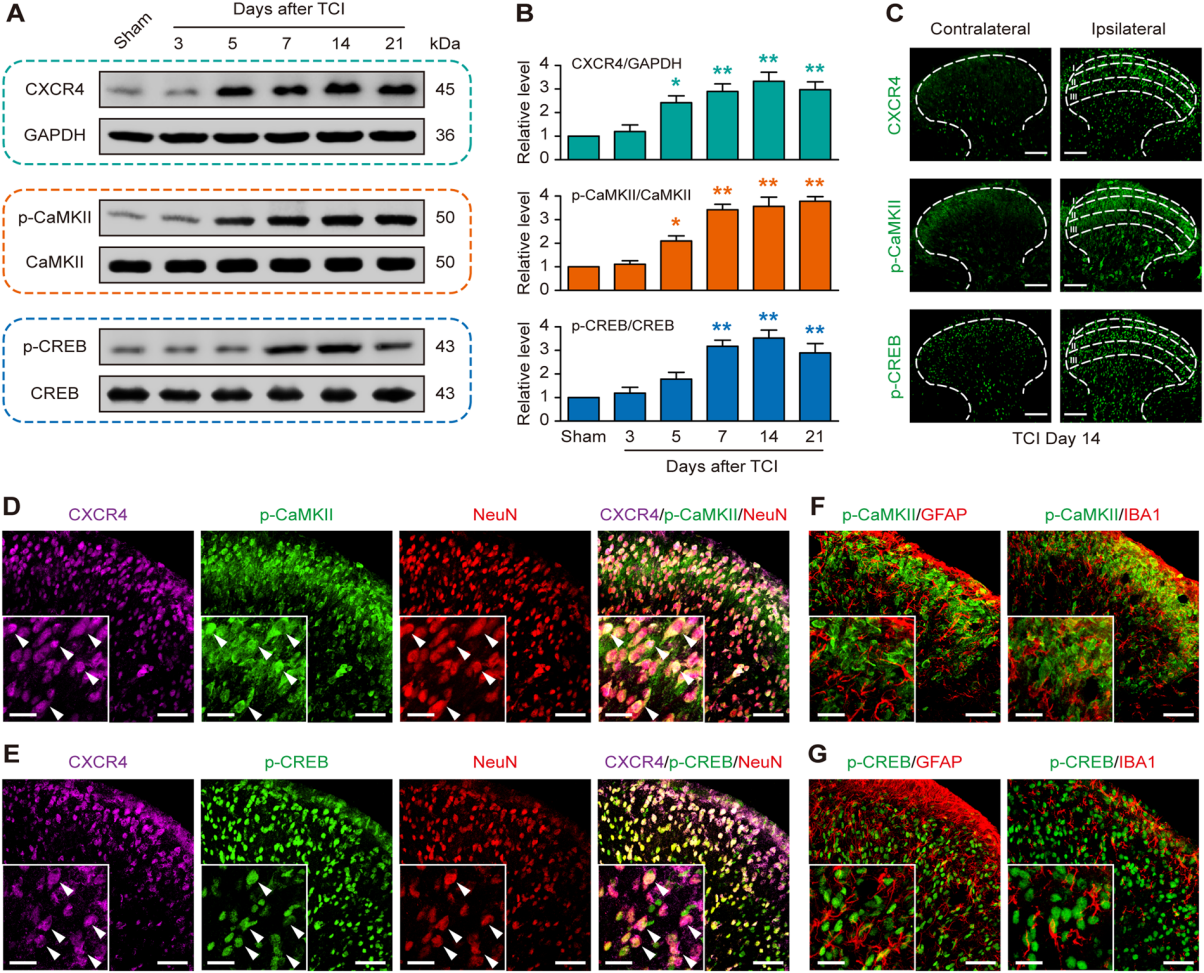
TCI increases the expression and co-localization of CXCR4, p-CaMKII and p-CREB in rat spinal dorsal neurons. (**A** and **B**) Western blot showing the time course of CXCR4 (top row), p-CaMKII (middle row) and p-CREB (bottom row) expression after TCI. (**A**) representative bands; (**B**) quantitative data. *P < 0.05, **P < 0.01 vs sham group; n = 4 for each group. (**C**) Immunofluorescence staining showing the expression and distribution of CXCR4 (top row), p-CaMKII (middle row) and p-CREB (bottom row) in the spinal cord dorsal horn. (**D–G**) Double and triple immunofluorescence staining showing cellular co-localization of CXCR4, p-CaMKII/p-CREB and cell markers in TCI rat spinal cord. CXCR4 (violet) co-localized with p-CaMKII (green, **D**) and p-CREB (green, **E**) in neurons (NeuN, red). Meanwhile, p-CaMKII (green, **F**) and p-CREB (green, **G**) did not co-express with astrocytes (GFAP, red) or microglial cells (IBA1, red). L4-L6 spinal tissues were taken on day 14 after TCI. Original magnification: 200× (**C**), 400× (**D–G**), and 1600 × (inserts in **D–G**); scale bar: 100 μm (**C**), 50 μm (**D–G**), and 20 μm (inserts in **D–G**).

Tumor-induced chemokine changes in the spinal dorsal horn are essential for the generation of cancer pain^20–22^. TCI induced a long-lasting up-regulation of CXCR4 protein in the spinal cord of the affected limb, which began on day 5 and was maintained until day 21, the last test day (Fig. 1A and B). These results are consistent with our previous studies^11^. As mentioned above, CaMKII/CREB signaling may be an important downstream pathway of CXCR4 and participate in CXCR4-mediated CIBP progression. Therefore, we further examined the time-course expression of p-CaMKII and p-CREB in the spinal cord of TCI and sham rats. Figure 1A and B showed that the p-CaMKII and p-CREB proteins increased in a time-dependent manner in the spinal cord, beginning on day 5 or 7 following TCI and remained at a high level until day 21. The total protein levels of CaMKII and CREB did not alter in sham or TCI rats throughout the detected time points. The immunofluorescence results also revealed increased expression of CXCR4, p-CaMKII and p-CREB in the ipsilateral spinal dorsal horn on day 14 after TCI, but no increase in the contralateral spinal dorsal horn (Fig. 1C).

Triple immunostaining demonstrated that CXCR4 predominantly co-localized with p-CaMKII (Fig. 1D) and p-CREB (Fig. 1E) in neurons (NeuN) on day 14 after TCI. Double immunostaining revealed that p-CaMKII (Fig. 1F) and p-CREB (Fig. 1G) did not co-express with astrocytes (GFAP) or microglial cells (IBA1). These findings suggest a functional relationship between CXCR4 and CaMKII/CREB pathway in spinal neurons under CIBP situations.

### Role of CXCR4 in nociceptive behaviors and CaMKII/CREB activation in cancer pain rats

**Figure 2:**
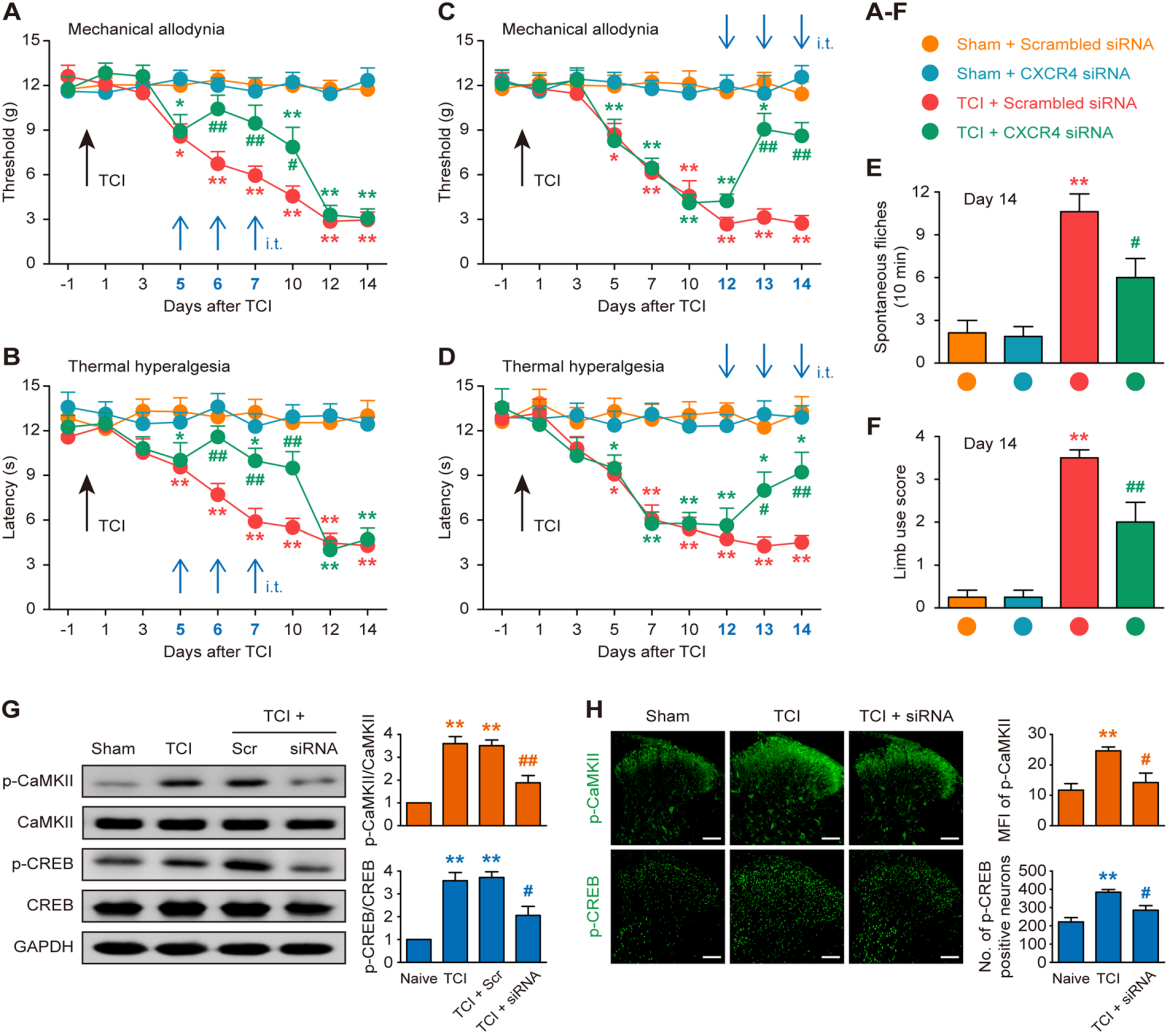
Intrathecal injection of CXCR4 siRNA attenuates TCI-induced cancer pain and up-regulation of p-CaMKII and p-CREB expression. Intrathecal injection of CXCR4 siRNA significantly attenuated TCI-induced mechanical allodynia (**A** and **C**), thermal hyperalgesia (**B** and **D**), spontaneous flinches (**E**) and limb uselessness (**F**) in bone cancer rats. CXCR4 siRNA (5 μg/10 μl, i.t.) or scrambled siRNA (negative control, 5 μg/10 μl, i.t.) was administered once daily on days 5, 6 and 7 (**A** and **B**) or days 12, 13 and 14 (**C–F**) after TCI. Behavioral tests were performed 12 hours after each injection. *P < 0.05, **P < 0.01 vs sham + scrambled siRNA group; ^#^ *P* < 0.05, ^##^ *P* < 0.01 vs TCI + scrambled siRNA group; n = 8 for each group. (**G**) Western blot analysis showing the inhibitory effect of CXCR4 siRNA on TCI-induced over-expression of p-CaMKII and p-CREB on day 14 after TCI. CXCR4 siRNA (siRNA, 5 μg/10 μl) or scrambled siRNA (Scr, 5 μg/10 μl) were administered once daily from days 12 to 14. L4-L6 spinal tissues were collected 12 hours after the last injection. **P < 0.01 vs sham group; ^#^ *P* < 0.05, ^##^ *P* < 0.01 vs TCI group; n = 4 for each group. (**H**) Immunofluorescence analysis showing inhibitory effect of CXCR4 siRNA on TCI-induced increase in mean fluorescence intensity (MFI) of p-CaMKII and the number (no.) of p-CREB-positive neurons on day 14 after TCI. CXCR4 siRNA (5 μg/10 μl) was administered once daily from days 12 to 14. L4-L6 spinal tissues were collected 12 hours after the last injection. **P < 0.01 vs sham group; ^#^ *P* < 0.05 vs TCI group; n = 4 for each group. Original magnification: 200×; scale bar: 100 μm.

The above findings implicated CXCR4 in spinal cord neurons in the pathogenesis of CIBP. Therefore, we further investigated the role of spinal CXCR4 in the initiation and maintenance of CIBP by knockdown of CXCR4 using siRNA. The knockdown effect of CXCR4 siRNA was confirmed using western blot with a CXCR4 antibody (Supplementary Fig. 1). We first examined the analgesic effect of CXCR4 siRNA on TCI-induced pain behaviors. The behavioral results showed that TCI progressively promoted prominent pain behaviors, which were represented as reductions in the paw mechanical withdrawal threshold (indicating mechanical allodynia) and paw thermal withdrawal latency (indicating thermal hyperalgesia) from post-operative days 5 to 14, as well as induction of spontaneous flinches and loss of limb use (indicating spontaneous pain) on day 14. However, CXCR4 siRNA (5 μg per injection, i.t., once daily for three consecutive days on days 5, 6 and 7) remarkably attenuated the initiation of mechanical allodynia and thermal hyperalgesia at the early phase of CIBP (Fig. 2A and B). Similarly, CXCR4 siRNA (5 μg per injection, i.t., once daily for three consecutive days on days 12, 13 and 14) reversed the established mechanical allodynia and thermal hyperalgesia (Fig. 2C and D) as well as spontaneous pain and loss of limb use (Fig. 2E and F) at the late phase of CIBP. Scrambled siRNA had no effect on pain hypersensitivity throughout the detected time-course. These results demonstrate that spinal CXCR4 is functionally up-regulated after TCI and plays a nociceptive role in the pathological progress of CIBP.

We continued to examine whether the neuronal CaMKII/CREB pathway was a downstream pathway of CXCR4 in spinal neurons, because CXCR4 was evidently co-expressed with p-CaMKII or p-CREB and the neuronal marker NeuN (Fig. 1D and E). The western blot and immunofluorescence results demonstrated that repeated administration of CXCR4 siRNA (5 μg per injection, i.t., once daily for three consecutive days on days 12, 13 and 14 after TCI) apparently reduced the up-regulation of p-CaMKII and p-CREB in the spinal cord on day 14 (Fig. 2G and H). These results explicitly indicate that CaMKII/CREB pathway is a functional downstream target of CXCR4 in spinal neurons under cancer pain conditions.

### Role of CaMKII in nociceptive behaviors and CREB activation in cancer pain rats

**Figure 3:**
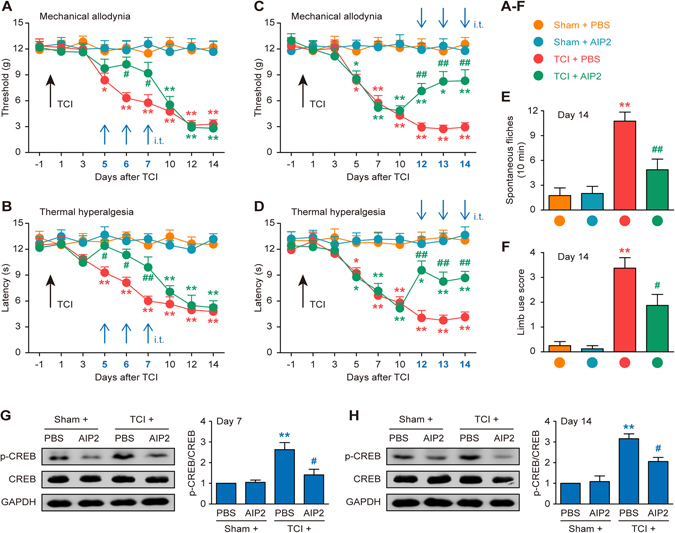
Intrathecal injection of CaMKII specific-inhibitor AIP2 attenuates TCI-induced cancer pain and p-CREB up-regulation. Intrathecal injection of AIP2 significantly delayed or attenuated TCI-induced mechanical allodynia (**A** and **C**), thermal hyperalgesia (**B** and **D**), spontaneous flinches (**E**) and limb uselessness (**F**) in bone cancer rats. AIP2 (10 μM/10 μl, i.t.) or PBS (vehicle control, 10 μl, i.t.) was administered once daily on days 5, 6 and 7 (**A** and **B**) or days 12, 13 and 14 (**C–F**) after TCI. Behavioral tests were performed 4 hours after each injection. *P < 0.05, **P < 0.01 vs sham + PBS group; ^#^ *P* < 0.05, ^##^ *P* < 0.01 vs TCI + PBS group; n = 8 for each group. (**G** and **H**) Inhibitory effect of AIP2 on p-CREB expression in TCI rats on day 7 or day 14. AIP2 (10 μM/10 μl, i.t.) or PBS (10 μl, i.t.) were administered once daily on days 5, 6 and 7 (**G**) or days 12, 13 and 14 (**H**) after TCI. L4-L6 spinal tissues were collected 4 hours after the last injection. **P < 0.01 vs sham + PBS group; ^#^ *P* < 0.05 vs TCI + PBS group; n = 4 for each group.

We further investigated the anti-nociceptive effect of CaMKII inhibitor autocamtide-2-related inhibitory peptide (AIP2), a CaMKII-specific peptide analogue, on the generation of CIBP hypersensitivity. The behavioral results showed that repetitive delivery of AIP2 (10 μM per injection, i.t., once daily for three consecutive days on days 5, 6 and 7 after TCI) distinctly delayed the progression of mechanical allodynia and thermal hyperalgesia after TCI (Fig. 3A and B). AIP2 (10 μM per injection, i.t., once daily for three consecutive days on days 12, 13 and 14 after TCI) also alleviated existing CIBP-like behaviors (Fig. 3C–F). These results reveal that spinal CaMKII/CREB pathway is functionally activated after TCI and plays a role in the pathogenesis of CIBP.

We then examined whether CaMKII stimulated CREB activation in spinal neurons under cancer pain conditions. The western blot results revealed that AIP2 (10 μM per injection, i.t., once daily for three consecutive days on days 5, 6 and 7 or days 12, 13 and 14) significantly suppressed TCI-induced CREB phosphorylation in the spinal cord on days 7 and 14 (Fig. 3G and H).

### Spinal injection of SDF-1 induces CXCR4/PLC-dependent CaMKII/CREB activation in naive rats

**Figure 4:**
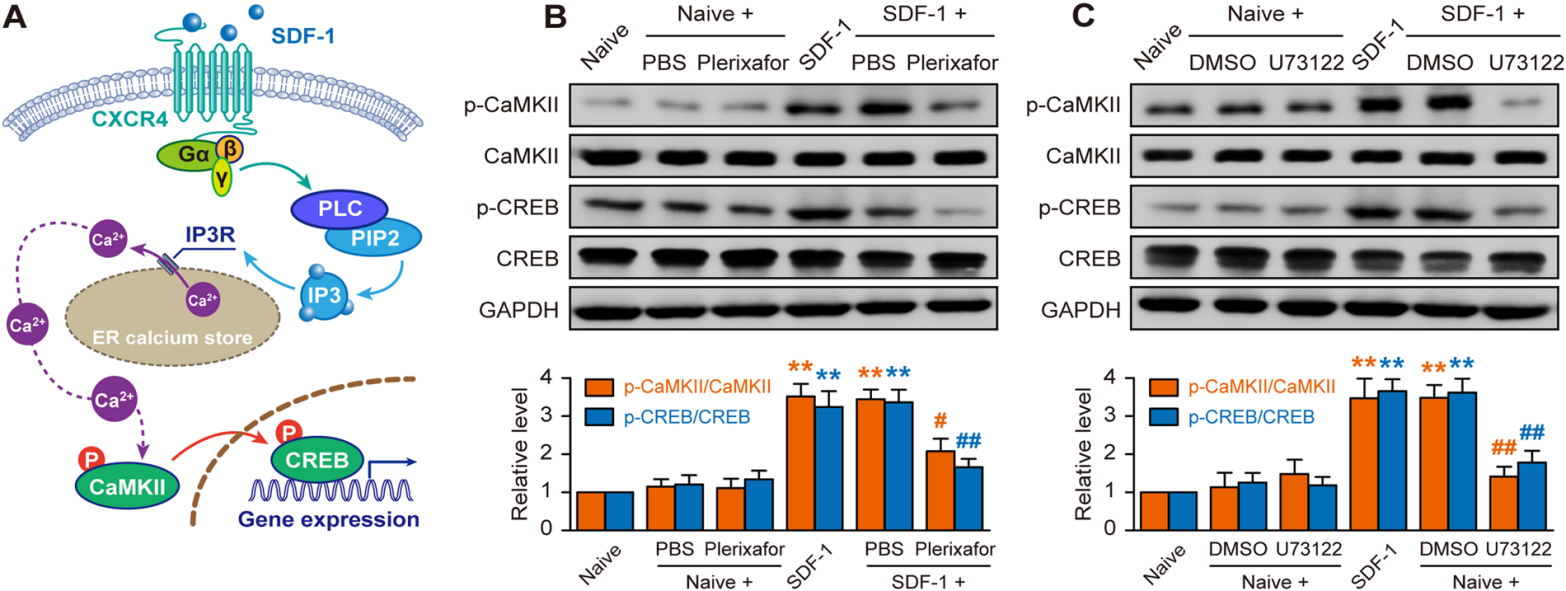
Exogenous SDF-1 induces spinal CaMKII/CREB phosphorylation through CXCR4 and PLC in naive rats. (**A**) A schematic overview of the CaMKII/CREB pathway activated by CXCR4 and the related intracellular signaling mediators in spinal neurons. (**B** and **C**) Intrathecal injection of exogenous SDF-1 (5 μg/10 μl) markedly up-regulated the expression of p-CaMKII and p-CREB in the spinal cord. Post-administration of CXCR4 inhibitor Plerixafor (10 μg/10 μl, **B**) or PLC inhibitor U73122 (10 nM/10 μl, **C**), 30 min after SDF-1 injection, could notably suppress SDF-1-induced over-expression of p-CaMKII and p-CREB. L4-L6 spinal tissues were collected 8 hours after SDF-1 injection. **P < 0.01 versus naive group; ^#^ *P* < 0.05, ^##^ *P* < 0.01 vs SDF-1 group; n = 4 for each group.

As we speculated, CXCR4, as a typical member of GPCRs, might activate CaMKII/CREB pathway through PLC-mediated intracellular Ca^2+^ release from the ER^23, 24^. Figure 4A summarizes the mechanism underlying the contribution of CXCR4 to CaMKII/CREB activation. We here examined the relationship between CXCR4 and the intracellular PLC/CaMKII/CREB signaling pathway using i.t. injection of exogenous SDF-1 in naive rats. The western blot results revealed that the p-CaMKII and p-CREB expression levels were increased 8 hr after a single i.t. administration of recombinant rat SDF-1 (5 μg) in naive rats, and i.t. administration of CXCR4-specific inhibitor Plerixafor (AMD3100, 10 μg) 30 min after SDF-1 injection significantly suppressed this SDF-1-induced increase in p-CaMKII and p-CREB expression (Fig. 4B). Furthermore, consistent with our hypothesis, i.t. administration of PLC-specific inhibitor U73122 (10 nM, 30 min after SDF-1 injection) also abrogated SDF-1-induced p-CaMKII and p-CREB over-expression in the spinal cord (Fig. 4C). These results demonstrate that CXCR4 and intracellular PLC mediate SDF-1-induced CaMKII/CREB activation in naive rats.

### CXCR4/PLC/CaMKII signaling is functionally involved in SDF-1-induced pain hypersensitivity

**Figure 5:**
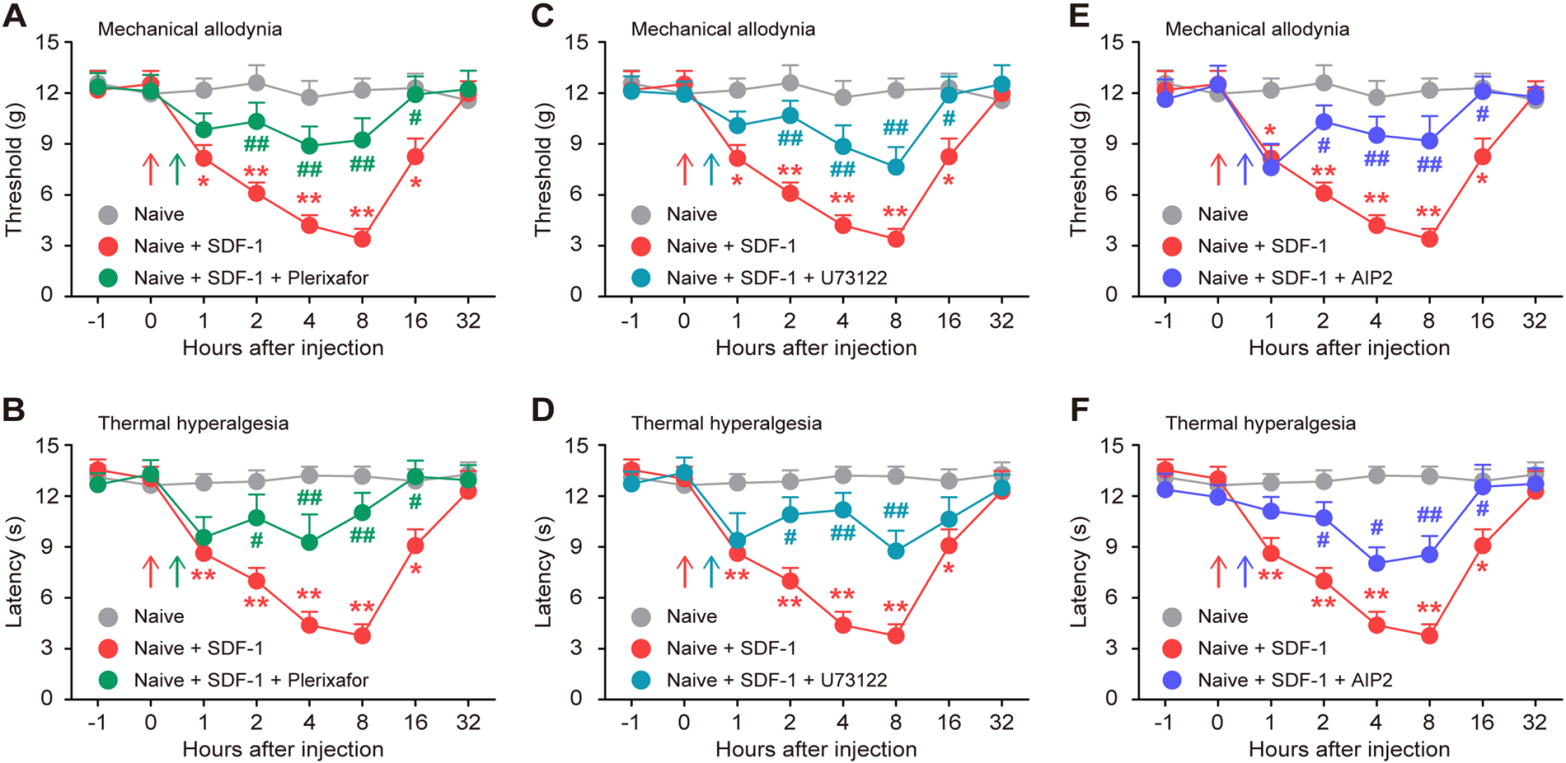
Spinal CXCR4-PLC-CaMKII pathway is involved in SDF-1-induced pain hypersensitivity. Intrathecal injection of exogenous SDF-1 (5 μg/10 μl) markedly induced mechanical allodynia (**A**,**C** and **E**) and thermal hyperalgesia (**B,D** and **F**) in a time-dependent manner. Post-administration of CXCR4 inhibitor Plerixafor (10 μg/10 μl, **A** and **B**), PLC inhibitor U73122 (10 nM/10 μl, **C** and **D**) or CaMKII inhibitor AIP2 (10 μM/10 μl, **E** and **F**), 30 min after SDF-1 injection, partially reversed SDF-1-induced nociceptive behaviors. The behavioral experiments between groups were performed at the same time points under the same experimental conditions. The same data of naive group and naive + SDF-1 group were rearranged and reused in A/C/E (threshold) and B/D/F (latency), respectively, to present the behavioral results more clearly and minimize the number of animals used. *P < 0.05, **P < 0.01 versus naive group; ^#^ *P* < 0.05, ^##^ *P* < 0.01 vs naive + SDF-1 group; n = 8 for each group.

We investigated the necessity of the PLC/CaMKII/CREB pathway in SDF-1-induced nociceptive sensitization. The behavioral tests showed that SDF-1 (5 μg, i.t.) produced time-dependent pain behaviors (mechanical allodynia and thermal hyperalgesia) within 1 hour, which were maintained for more than 16 hours in naive rats. However, i.t. administration of Plerixafor (10 μg, Fig. 5A and B), U73122 (10 nM, Fig. 5C and D) or AIP2 (10 μM, Fig. 5E and F) 30 min after SDF-1 injection partially prevented SDF-1-induced reductions of the paw mechanical withdrawal threshold and paw thermal withdrawal latency. Collectively, these findings demonstrate that spinal SDF-1/CXCR4 signaling is sufficient to induce pain hypersensitivity by activating the PLC-mediated CaMKII/CREB pathway in naive rats.

### Spinal CXCR4 facilitates the up-regulation of NMDAR1 in cancer pain rats

**Figure 6:**
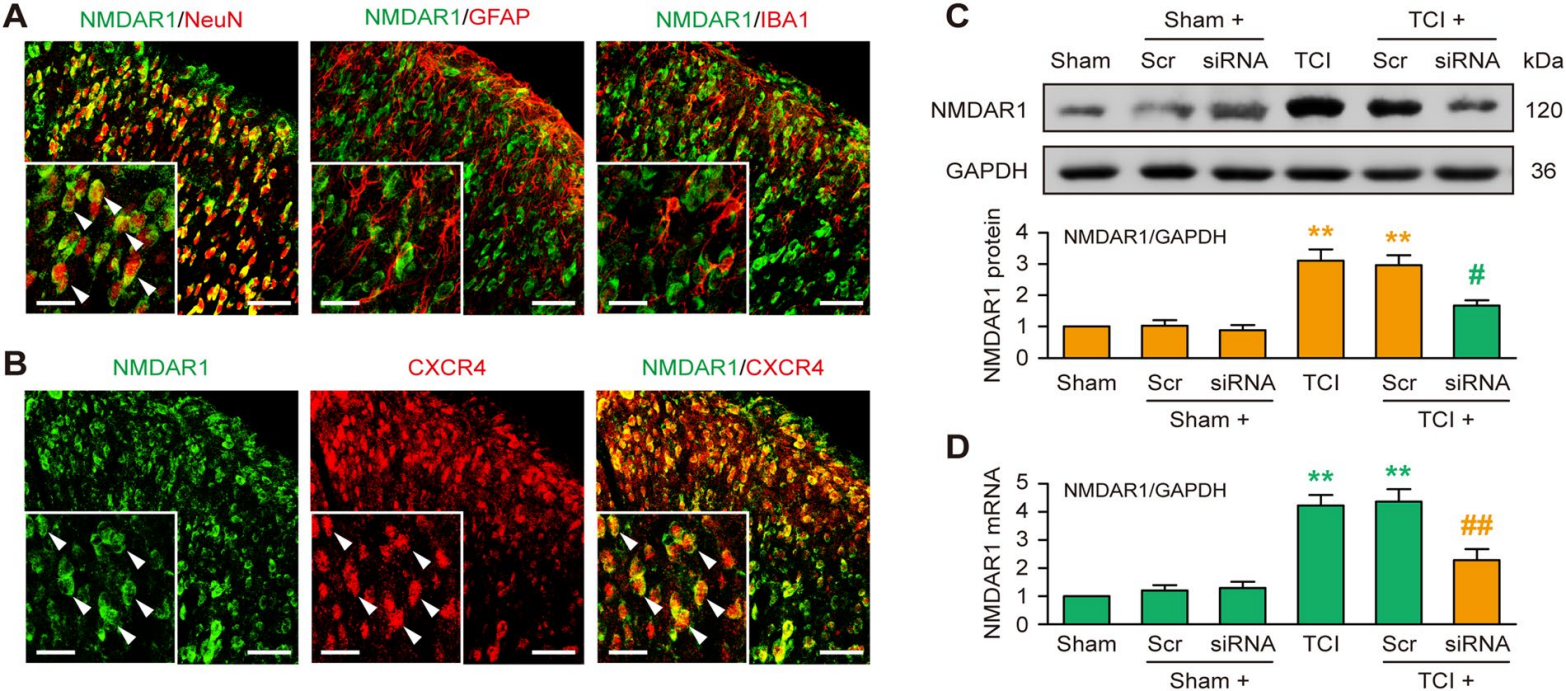
Intrathecal injection of CXCR4 siRNA suppresses TCI-induced up-regulation of NMDAR1 expression in spinal neurons. (**A**) Immunofluorescence staining showing that NMDAR1 (green) co-localized with neurons (NeuN, red), but not astrocytes (GFAP, red) or microglial cells (IBA1, red). (**B**) CXCR4 (red) co-expressed with NMDAR1 (green) in the spinal cord. Tissues were taken on day 14 after TCI. Original magnification: 400× (**A** and **B**), and 1600 × (inserts in **A** and **B**); scale bar: 50 μm (**A** and **B**), and 20 μm (inserts in **A** and **B**). (**C** and **D**) Western blot and RT-qPCR analyses showing the inhibitory effect of CXCR4 siRNA on TCI-induced over-expression of NMDAR1 protein (**C**) and mRNA (**D**) on day 14 after TCI. CXCR4 siRNA (siRNA, 5 μg/10 μl) or scrambled siRNA (Scr, 5 μg/10 μl) were administered once daily from days 12 to 14. L4-L6 spinal tissues were collected 12 hours after the last injection. **P < 0.01 vs sham group; ^#^ *P* < 0.05, ^##^ *P* < 0.01 vs TCI group; n = 4 for each group.

Activation of N-methyl-D-aspartate receptors (NMDARs) in the spinal cord is an essential mechanism of central sensitization and pain signal transmission^25^. NMDAR1 is a pivotal subunit of NMDARs and is transcriptionally regulated by CREB in a phosphorylation-dependent manner^26, 27^. Therefore, we investigated whether CXCR4 in spinal neurons modulated NMDAR1 expression after TCI. Figure 6A and B showed that NMDAR1 primarily co-expressed with neurons (NeuN), but not astrocytes (GFAP) or microglial cells (IBA1), and CXCR4 clearly co-localized with NMDAR1 on day 14 after TCI. The western blot and RT-qPCR data revealed that i.t. administration of CXCR4 siRNA (5 μg per injection, once daily for three consecutive days on days 12, 13 and 14 after TCI) significantly reduced the increased expression of the NMDAR1 protein and mRNA on day 14 after TCI (Fig. 6C and D). Collectively, these results suggest that spinal CXCR4 promotes the up-regulated expression of NMDAR1 at the transcriptional level in cancer pain situations.

## Discussion

The association of neuronal sensitization with intracellular signaling activation is the primary mechanism underlying chronic pain^28, 29^. Accumulating evidence suggests that chemokine receptors (e.g., CCR2, CXCR2 and CXCR3) contribute to neuronal sensitization in cancer pain generation^20, 22, 30^. Our previous study demonstrated an up-regulation of chemokine receptor CXCR4 in spinal neurons, and this up-regulation was involved in TCI-induced pain hypersensitivity^11^. However, the distinct neuronal mechanism of CXCR4 in the development and maintenance of CIBP was not clearly elucidated. The current study revealed some novel results. (1) TCI produced a persistent up-regulation of CXCR4, p-CaMKII and p-CREB expression in the spinal cord, and these proteins exhibited significant co-expression in neurons. (2) Repeated i.t. administration of CXCR4 siRNA suppressed TCI-induced nociceptive behaviors and CaMKII/CREB activation in the spinal cord. (3) Repeated i.t. administration of CaMKII-specific inhibitor AIP2 suppressed TCI-induced nociceptive behaviors and CREB activation. (4) A single i.t. injection of exogenous SDF-1 triggered time-dependent pain hypersensitivity and CaMKII/CREB activation in the spinal cord. (5) Post-administration of CXCR4 inhibitor Plerixafor, PLC inhibitor U73122, or CaMKII inhibitor AIP2 partially abolished SDF-1-induced pain behaviors and CaMKII/CREB activation. (6) TCI also increased the expression of NMDAR1 protein and mRNA, which localized in spinal neurons and co-localized with CXCR4 in the spinal cord. CXCR4 siRNA suppressed TCI-induced NMDAR1 up-regulation at the transcriptional level. Taken together, these findings suggest that chemokine receptor CXCR4 promotes CIBP sensitization and NMDAR1 up-regulation by activating the intracellular CaMKII/CREB pathway in spinal neurons.

CXCR4 has been shown to mediate the spinal nociceptive processing under chronic pain conditions, as i.t. administration of AMD3100, a potent and selective CXCR4 inhibitor, significantly alleviated various neuropathic pain^5, 31–33^, and opioid-induced hyperalgesia^34^. Our previous research demonstrated an up-regulation of CXCR4 after TCI in the spinal cord, and blockade of CXCR4 with AMD3100 significantly delayed and suppressed the induction and persistence of CIBP. Specific siRNAs were injected to knock down CXCR4 in the present study to avoid the pharmacological limitations of AMD3100. Repetitive i.t. administration of CXCR4 siRNA remarkably suppressed TCI-induced cancer pain behaviors (mechanical allodynia, thermal hyperalgesia, spontaneous flinching and loss of limb use), which is consistent with our previous findings^11^. It is noteworthy that accumulating evidence has revealed the over-expression of CXCR4 in the dorsal root ganglia (DRG) under peripheral inflammatory or neuropathic pain states, which is co-expressed with A-type (NF-200-positive) neurons and C-type peptidergic (substance P-positive) or non-peptidergic (IB4-positive) neurons^7, 35^, as well as GS, or GFAP-positive satellite glial cells^7, 36^. Meanwhile, previous studies suggest the analgesic effect of CXCR4 antagonist AMD3100 in different pain models. For example, intraplantar injection of AMD3100 attenuated bee venom-induced inflammatory pain^35, 37^; i.t. application with AMD3100 suppressed the hypersensitivity of DRG neurons and trinitrobenzene sulfonic acid-induced abdominal pain^38^; intraperitoneal (i.p.) administration of AMD3100 reversed diabetic peripheral neuropathic pain^31^ and opioid-induced hyperalgesia^34^; i.t. and/or i.p. delivery of AMD3100 attenuated neuropathic pain behaviors following spared nerve injury^7^ or spinal nerve ligation^33^. Therefore, in our present study, we cannot exclude the possibility that CXCR4 in DRG could be suppressed by i.t. siRNA injection and might participate in the alleviation of CIBP. Considering the present research was just focused on the spinal mechanism of CXCR4 underlying CIBP generation, we did not explore whether CXCR4 in DRG is involved in the development and maintenance of CIBP. However, we have to admit that the pain behavioral results may be partially affected by the CXCR4 expression in DRG, based on the existing data, although the conclusions may not be changed.

CXCR4 exerts different biological effects by initiating different intracellular signaling pathways in different cell types^39^. Studies have proved that CXCR4 in the DRG and spinal cord plays an essential role in the pathogenesis of neuropathic pain and opioid tolerance through various downstream pathways, such as MAPKs (ERK, JNK and p38)^7, 40^, Akt^37^, and SFK^41^. This study examined the CaMKII/CREB pathway because it is important for the modulation of spinal central sensitization in chronic pain. Accumulating evidence suggests that CaMKII/CREB is functionally implicated in animal models of inflammatory pain^42^, neuropathic pain^43^ and visceral pain^44^. Therefore, we herein investigated whether CaMKII/CREB was stimulated in the bone cancer state and contributed to pain sensitization. The current results demonstrated that TCI induced CaMKII phosphorylation at Thr186 and CREB phosphorylation at Ser133 in a time-dependent manner in spinal neurons, and this up-regulation was accompanied by mechanical allodynia, thermal hyperalgesia, spontaneous flinches and loss of limb use. While i.t. treatment with CaMKII-specific inhibitor AIP2 significantly prevented and alleviated CIBP in the early and late stages of the TCI model, and decreased TCI-induced CREB phosphorylation. These results are consistent with previous studies that demonstrated a role of CaMKII/CREB in bone cancer pain^45, 46^ and the anti-hyperalgesic effect of AIP2 in various pain models^47–49^. Furthermore, CaMKII is a specific type of calcium-dependent signaling, which is stimulated after Ca^2+^ influx through NMDARs during synaptic plasticity^50, 51^. CXCR4 is a conventional GPCR that also elicits Ca^2+^ influx from the ER via the PLC pathway^52, 53^. Therefore, we hypothesized that CXCR4 would directly regulate the CaMKII/CREB pathway to induce chronic nociceptive hypersensitivity. Notably, immunofluorescence revealed that CXCR4 primarily distributed in p-CaMKII/p-CREB-positive neurons in CIBP rats. Intrathecal injection of CXCR4 siRNA significantly inhibited TCI-induced up-regulation of p-CaMKII and p-CREB in the spinal cord. Exogenously delivered SDF-1 produced over-expression of p-CaMKII and p-CREB in naive rats, which were partially reversed by CXCR4 blockade with Plerixafor or PLC blockade with U73122. The separate inhibition of CXCR4, PLC or CaMKII also reversed SDF-1-induced pain behaviors. However, Plerixafor and U73122 almost completely blocked up-regulation of p-CaMKII and p-CREB, but these agents did not completely block the pain hypersensitivity induced by SDF-1, which is reasonably inconsistent. The following reasons likely explain these results. (1) CXCR4 is a G protein-coupled receptor that likely regulates many pain-related signal pathways in pain processing. CaMKII/CREB is an important factor in at least one of these pathways. Therefore, other intracellular molecular mechanisms may also participate in CXCR4-mediated pain sensation. (2) CXCR4 is located in spinal neurons and glial cells, but CaMKII/CREB is a purely neuronal mechanism of CXCR4-mediated pain sensation. SDF-1 injection may simultaneously induce the activation of astrocytes and microglial cells in the spinal cord, which may also drive nociceptive behaviors. (3) Plerixafor or U73122 was administered 30 min after SDF-1 injection. Therefore, Plerixafor or U73122 may not completely reverse SDF-1-mediated behavioral changes. Taken together, these findings demonstrated for the first time that CaMKII/CREB activation in spinal neurons is an important neuronal mechanism of CXCR4 signaling to pain sensitization in naive and cancer pain rats.

CREB is a major transcriptional factor in the orchestration of synaptic plasticity and chronic pain development by activating pain-related gene expression^54^. A recent study showed that a decrease in CREB-mediated transcriptional activity attenuated bone cancer-evoked pain behaviors and reduced CREB-target gene expression of the N-methyl-D-aspartate receptor subunit 2B (NMDAR2B)^46^. Our current research focused on NMDAR1, another subunit of NMDARs, which is also under transcriptional regulation of CREB, and this subunit is essential for central sensitization and nociceptive transmission^26, 27^. A previous study revealed that NMDAR1 mRNA expression in the spinal cord and DRG increased significantly in cancer pain rats^55^. The present study investigated the regulatory effect of CXCR4 in NMDAR1 expression. As expected, NMDAR1 protein and mRNA expression were up-regulated to some degree, and the expression was predominantly distributed in neurons and co-expressed with CXCR4 after TCI. Knock down of CXCR4 using repeated i.t. CXCR4 siRNA administration partially suppress TCI-induced NMDAR1 up-regulation at the transcriptional level. Thus, the current findings at least partially demonstrated that spinal NMDAR was critically involved in CXCR4 signaling-mediated cancer pain. Furthermore, NMDAR activation is primarily dependent on phosphorylation in chronic pain states, and previous studies reported that spinal expression of p-NMDAR1 and p-NMDAR2B was up-regulated in cancer pain rats^56, 57^. However, whether CXCR4 signaling increases the phosphorylation of NMDAR1 and NMDAR2B during the progression of CIBP requires further investigation.

In summary, our findings suggest that CXCR4 is a potent molecular signaling mechanism in the pathogenesis of CIBP and a potent therapeutic target for cancer pain treatment. Furthermore, the spinal CaMKII/CREB pathway is a critical downstream target for CXCR4-mediated neuronal sensitization and cancer pain hypersensitivity.

## Methods

### Animals

Adult female Sprague-Dawley rats (body weight 180–220 g) were purchased from Shanghai Experimental Animal Center of Chinese Academy of Sciences. Rats were housed under a constant 12 hr light/dark cycle (lights on from 08:00 to 20:00) at 23 ± 1 °C with food and water available *ad libitum*. The animals were housed at least three days for acclimation before initiating experimental protocols. The Institutional Animal Care and Use Committee of Xuzhou Medical University and Fudan University approved all animal procedures, which were designed to minimize the number of animals used and their suffering. The experimental protocols were consistent with the NIH Guide for the Care and Use of Laboratory Animals and the IASP Ethical Issues for pain research^58^.

### Model of cancer-induced bone pain

The surgical procedure was performed as described in our and other previous studies^11, 59^. Briefly, rats were anesthetized with pentobarbital sodium (50 mg/kg i.p.). The superficial skin of right hind tibia was shaved and disinfected with 75% (v/v) ethanol. Prepared Walker 256 mammary gland carcinoma cells (1 × 10^5^ cells/μl, 5 μl) were slowly injected into the tibial cavity using a microinjection syringe. The syringe needle was withdrawn, and the injection site was sealed with bone wax to prevent the tumor cells from leaking outside of the bone hole. Sham surgery (for control) used a similar protocol and injected 5 μl sterile PBS instead of tumor cells into the right tibia.

### Drugs and administration

Highly specific and cell permeable CaMKII inhibitor AIP2 (autocamtide-2-related inhibitory peptide, myristoylated) was purchased from Enzo Life Sciences (ALX-151-030-MC05, Farmingdale, NY, USA). CXCR4 inhibitor Plerixafor (AMD3100, S8030) and PLC inhibitor U73122 (S8011) were purchased from Selleck Chemicals (Shanghai, China). Rat recombinant SDF-1 (CXCL12) was purchased from ProSpec (CHM-354, East Brunswick, NJ, USA). Drugs were dissolved in sterilized PBS and delivered at a volume of 10 μl into cerebral spinal fluid via lumbar puncture. Intrathecal (i.t.) injection was performed using a 25-μl Hamilton syringe with a 30-gauge needle into the L5-6 interspace. The injection needle was left in place for 10 sec after each injection. Animals with signs of motor dysfunction were excluded from the experiments. All doses of drugs were based on preliminary experiments. The doses of each drug and treatment time points are presented in the figure legends.

### Small interfering RNA against CXCR4

Small interfering RNA (siRNA) knockdown of CXCR4 was obtained from GenePharma based on a GenBank search (Accession No. NM_022205.3). CXCR4 siRNA sequences were: 5′-GACUGGUACUUUGGGAAAUTT-3′ and 5′-AUUUCCCAAAGUACCAGUCTT-3′. A scrambled sequence (5′-GUAGCAGGGCA UGUAUUUATT-3′ and 5′-UAAAUACAUGCCCUGCUACTT-3′) was designed as a negative control. The siRNA was mixed with polyethyleneimine (PEI; 1 μg siRNA in 0.18 μl PEI; Sigma-Aldrich, St Louis, MO, USA) prior to intrathecal injection to increase cell membrane penetration^60^. For the bone cancer rats, siRNA (5 μg) was i.t. administered at days 5, 6, and 7 or days 12, 13, and 14 after TCI. The knockdown effect of CXCR4 was evaluated using western blot.

### Behavioral testing

*Mechanical allodynia* was determined via measurement of the paw mechanical withdrawal threshold (PWT) in response to von Frey filaments (Aesthesio®, Danmic Global, San Jose, CA, USA) stimulation^61^. A series of filaments (0.4, 0.6, 1.4, 2, 4, 6, 8 and 15 g) were applied to the mid-plantar surface of rat hindpaw with a sustaining pressure to bend the filament for 5 sec or induce a paw withdrawal reflex within 5 sec. The Dixon’s up-down method was used to determine the 50% PWT. *Thermal hyperalgesia* was assessed via measurement of the paw thermal withdrawal latency in response to radiant heat stimulation generated by a Plantar Analgesia Meter (IITC Life Science, CA, USA)^62^. The radiant heat terminated when the rat withdrew its hindpaw or automatically at a 20-sec cutoff to prevent tissue damage. The thermal stimulus was delivered 3 times to each hindpaw with 10 min inter-trial interval. *Spontaneous flinches* were measured to evaluate spontaneous pain during a 10-min observation period^63^. *Limb use score* was measured to assess movement-evoked pain during ambulation on a scale of 0 to 4: 0 = normal use; 1 = slightly limping; 2 = clearly limping; 3 = no use of the limbs (partial); and 4 = no use of the limbs (complete)^64^.

### Western blot

The L4-6 segments of the lumbar spinal cord were isolated and homogenized in a RIPA lysis buffer containing 1% PMSF and a protease/phosphatase inhibitor cocktail (#5872, Cell Signaling Technology, Danvers, MA, USA). Protein concentration was determined using the BCA protein assay Kit (Thermo Scientific), and 20 μg of protein per lane were loaded and separated using 10% SDS-PAGE. Separated proteins were transferred onto a 0.22-μm PVDF membrane. The membranes were placed in a blocking solution that contained Tris-buffered saline with 0.1% Tween (TBST) and 5% non-fat dry milk for 1 hr at room temperature and incubated with primary antibodies overnight at 4 °C. Membranes were incubated with specific HRP-conjugated secondary antibodies. The following primary antibodies were used: goat anti-CXCR4 (1:500 NB100-716, Novus Biologicals, Littleton, CO, USA), rabbit anti-phospho-CaMKII (Thr286, 1:1000, sc-12886, Santa Cruz Biotechnology, Dallas, TX, USA), mouse anti-CaMKII (1:1000, #50049, Cell Signaling Technology), rabbit anti-phospho-CREB (Ser133, 1:1000, #9198, Cell Signaling Technology), rabbit anti-CREB (1:1000, #9197, Cell Signaling Technology), rabbit anti-NMDAR1 (1:1000, #5704, Cell Signaling Technology) and mouse anti-GAPDH (1:10000, 60004-1-Ig, Proteintech, Rosemont, IL, USA). Proteins were detected using Immobilon Western Chemiluminescent HRP Substrate (WBKLS0500, Millipore, Billerica, MA, USA) and ImageQuant LAS 4000 mini (GE Healthcare). Band intensity was quantified using Quantity One Analysis Software (Version 4.6.5, Bio-Rad Laboratories, Hercules, CA, USA). Protein band densities were measured and normalized to GAPDH density. The fold change of the control group was set as 1 for quantifications.

### Real-time quantitative PCR

Total RNA from L4-6 spinal segments was extracted using TRIzol® reagent (thermo scientific) following the manufacturer’s instructions. RNA was reverse transcribed into cDNA using an oligo (dT) primer, and the cDNA served as a template for the detection of gene content. The NMDAR1 primers were forward: 5′-CAGCCGTGAACGTGTGGAG-3′ and reverse: 5′-TGCTCTACCACTCTTTCTATCC-3′, and the GADPH primers were forward: 5′-GGGTGTGAACCACGAGAAAT-3′ and reverse: 5′-ACTGTGGTCATGAGCCCTTC-3′. RT-qPCR analysis was performed using the SYBR Premix Ex Taq kit (RR420A, Takara, Otsu, Japan) in a LightCycler® 480 Real-Time PCR System (Roche, Shanghai, China). All primers were synthesized and purchased from GenePharma (GenePharma, Suzhou, China). The PCR thermal cycle conditions were 95 °C for 3 min, followed by 40 cycles of 95 °C (12 sec), and 62 °C (40 sec). Each reaction was performed in triplicate. The expression of NMDAR1 mRNA was normalized to GAPDH using the 2−∆∆Ct method^27, 60^.

### Immunofluorescence

Rats were intracardially perfused with phosphate buffer saline (PBS) and 0.1 M PB containing 4% paraformaldehyde (PFA) under deep anesthesia (sodium pentobarbital, 60 mg/kg, i.p.). The L4-6 spinal segments were dissected and post-fixed in 4% PFA overnight at 4 °C, transferred and preserved in 0.1 M PB containing 30% sucrose at 4 °C for subsequent use. Spinal tissues were serially sliced into 30-μm thick sections using a cryostat and stored in 0.01 M PBS. The sections were blocked in 10% donkey serum for 2 hr at room temperature and incubated overnight at 4 °C with the following primary antibodies: goat anti-CXCR4 (1:200), rabbit anti-phospho-CaMKII (Thr286, 1:500), rabbit anti-phospho-CREB (Ser133, 1:1000), rabbit anti-NMDAR1 (1:200, 21287, Signalway Antibody, College Park, MD, USA), mouse anti-NeuN (neuronal nuclear marker, 1:1000, MAB377, Millipore), mouse anti-GFAP (astrocyte marker, 1:1000, #3670, Cell Signaling Technology), and goat anti-IBA1 (microglia marker, 1:1000, ab5076, Abcam, Cambridge, MA, USA). Free-floating sections were washed 3 times for 10 min in 0.01 M PBS containing 0.3% Triton X-100 and incubated for 2 hr at room temperature with the corresponding Alexa Fluor® 405, 488, 594-conjugated secondary antibodies (1:1000, Invitrogen, Carlsbad, CA, USA) overnight at 4 °C. Stained sections for double immunostaining were incubated for a second time using the same procedures described above. Fluorescent images were captured using a confocal scanning laser microscope (FV1000, Olympus, Tokyo, Japan), and images are shown as merged Z-stack projections consisting of ~10 optical slices. The mean fluorescence intensity (MFI) of p-CaMKII immunofluorescence and the number of p-CREB immunopositive neurons in the entire superficial dorsal horn, included 16 spinal cord sections from 4 rats (4 sections from each rat) in each group, were measured by ImageJ software^60, 65^.

### Statistical analysis

All data are expressed as mean as the means ± SEM, and all statistical analysis were performed using GraphPad Prism 5 (GraphPad Software, San Diego, CA, USA). Alterations of detected mRNA and protein expression were tested using one-way analysis of variance (ANOVA) followed by Bonferroni *post hoc* test. Changes in nociceptive behaviors over time between groups were tested using two-way ANOVA with repeated measures followed by Bonferroni *post hoc* test. Significant differences were considered significant if *P* < 0.05.

## Electronic supplementary material

Supplementary Information
