# Supplementary material for: Chemokine receptor CXCR4 regulates CaMKII/CREB pathway in spinal neurons that underlies cancer-induced bone pain
Source: Sci Rep. 2017 Jun 21;7:4005. doi: 10.1038/s41598-017-04198-3 (PMC5479786; doi:10.1038/s41598-017-04198-3)
Supplement: Supplementary file 1 — Supplementary Information [file 41598_2017_4198_MOESM1_ESM.pdf]

Supplementary Figure

Chemokine receptor CXCR4 regulates CaMKII/CREB pathway in spinal neurons that underlies cancer-induced bone pain

Xue-Ming Hu[1,2,*], Hui Zhang[2,*], Heng Xu[1,2,*], Hai-Long Zhang[2], Li-Ping Chen[1,2], Wen-Qiang Cui[3], Wei Yang[3] & Wen Shen[1,2,†]

[1]*Department of Pain Medicine, Affiliated Hospital of Xuzhou Medical University, Xuzhou, Jiangsu 221002, China.*

[2]*Jiangsu Province Key Laboratory of Anesthesiology and Jiangsu Province Key Laboratory of Anesthesia and Analgesia Application Technology, Xuzhou Medical University, Xuzhou, Jiangsu 221002, China.*

[3]*Department of Integrative Medicine and Neurobiology, Academy of Integrative Medicine, School of Basic Medical Sciences, Fudan University, Shanghai 200032, China.*

**These authors contributed equally to this work.*

[†]Correspondence and requests for materials should be addressed to W. Shen (email: shenwen19962@163.com).

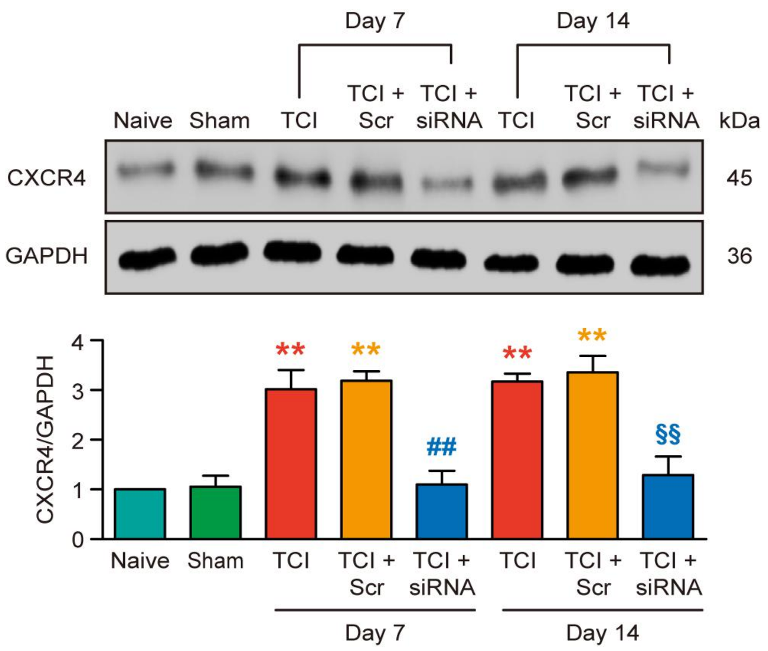

Supplementary Figure 1: Knockdown effect of siRNA on CXCR4 protein expression on day 7 or day 14 in TCI rats. CXCR4 siRNA (siRNA, 5 μg/10 μl, i.t.) or scrambled siRNA (Scr, 5 μg/10 μl, i.t.) was administered once daily on days 5, 6 and 7 or days 12, 13 and 14 after TCI. L4-L6 spinal tissues were collected 12 hours after the last injection on day 7 or day 14, respectively. $^{**}P < 0.01$ vs naive group; $^{\#\#}P < 0.01$ vs TCI Day 7 group; $^{\S\S}P < 0.01$ vs TCI Day 14 group; n = 4 for each group.
